# Ferulic Acid Esterase Producing *Lactobacillus johnsonii* from Goat Feces as Corn Silage Inoculants

**DOI:** 10.3390/microorganisms10091732

**Published:** 2022-08-27

**Authors:** Estefania Andrada, Mónica Adriana Mechoud, María Claudia Abeijón-Mukdsi, Elsa Patricia Chagra Dib, Santiago Cerviño, Adriana Perez Chaia, Roxana Beatriz Medina

**Affiliations:** 1 Centro de Referencia para Lactobacilos, Consejo Nacional de Investigaciones Científicas y Técnicas, San Miguel de Tucumán, Tucumán T4000ILC, Argentina; 2 Facultad de Agronomía y Zootecnia, Universidad Nacional de Tucumán, San Miguel de Tucumán, Tucumán T4000ACS, Argentina; 3 Estación Experimental Agropecuaria Salta, Instituto Nacional de Tecnología Agropecuaria, Cerrillos, Salta A4403XAA, Argentina; 4 Zona Valles Calchaquíes, Subsecretaría de Agricultura Familiar, Campesina e Indígena, Ministerio de Agricultura, Ganadería y Pesca, San Miguel de Tucumán, Tucumán T4000GBD, Argentina

**Keywords:** silage inoculants, ferulic acid esterase, lactic acid bacteria, *Lactobacillus johnsonii*

## Abstract

Ferulic acid esterase (FAE+)-producing lactobacilli are being studied as silage inoculants due to their potential of increasing forage fiber digestibility. In this work, three FAE+ *Lactobacillus (L.) johnsonii* strains were isolated from caprine feces and characterized according to their potential probiotic characteristics and as silage inoculants. *Limosilactobacillus fermentum* CRL1446, a human probiotic isolated from goat cheese, was also included in the experiments as a potential silage inoculant. FAE activity quantification, probiotic characterization, and growth in maize aqueous extract indicated that *L. johnsonii* ETC187 might have a better inoculant and probiotic aptitude. Nevertheless, results in whole-corn mini silos indicated that, although acid detergent fiber (ADF) was significantly reduced by this strain (3% compared with the uninoculated (UN) group), *L. johnsonii* ETC150 and CRL1446 not only induced similar ADF reduction but also reduced dry matter (DM) loss (by 7.3% and 6.5%, respectively) compared with the UN group. Additionally, CRL1446 increased in vitro DM degradability by 10%. All treatments reduced gas losses when compared with the UN group. The potential probiotic features of these strains, as well as their beneficial impact on corn fermentation shown in this study, encourage further studies as enhancers in animal production.

## 1. Introduction

Conservation of forage through lactic fermentation is a broadly used method in ruminant production systems. Lactic acid bacteria (LAB) inoculants are a useful and convenient tool to address several challenges in forage preservation, such as low acidification rate and aerobic spoilage. Increasing the fiber digestibility of forages during the fermentation process is an opportunity to maximize feed conversion by animals, and it was accomplished in some cases by the addition of additives consisting of purified fungal or bacterial fibrolytic enzymes, especially cellulases and hemicellulases [1]. The addition of purified enzymes is a less convenient approach in production systems when compared with LAB inoculants, as they can be detrimental to agricultural machinery and comparatively more expensive. The efficiency of fibrolytic enzymes is not always satisfactory owing to the lack of effect, or a negative effect, on the dry matter (DM) and neutral detergent fiber (NDF) degradability of forage, despite the improvements in silage fermentation [1,2,3]. The reason could be that cellulase and hemicellulase action is limited to more digestible components during ensiling [4]. 

Ferulic acid esterases (FAEs) or cinnamoyl esterases are hemicellulose-acting enzymes that contribute to hydrolyzing dietary fiber, improving its ruminal digestibility [5]. Within the main components of digestible fiber in forage are arabinoxylans and glucuronoarabinoxylans. They are linked via an ester bond to ferulic acid (FA) or to each other with other hydroxycinnamic acids (HAs), such as caffeic, sinapic, and p-coumaric acids. FA is the most abundant HA in plant cell walls, where it is esterified. In this state, it plays an important role in inhibiting fiber digestion in ruminants by interfering with the binding of rumen bacteria to the plant cell wall. FA also forms ester bonds with lignin, further reducing fiber digestibility. Therefore, the hydrolysis of these ester linkages by FAEs is expected to increase ruminal fiber degradation, energy supply, and animal productivity [6,7,8]. Corn is a ferulate-rich crop [9], and even small changes in the ester-linked FA content are expected to have an impact on ruminal degradability [10]. Likewise, FAEs favor the fermentation of vegetable fiber by epiphytic microorganisms, improving silage conservation. 

Besides its implication in fiber digestibility, FA is a known beneficial compound for human and animal health due to its antioxidant and anti-inflammatory activities, among many other functions [11]. For instance, previous results showed that oral administration of the probiotic FAE+ *Limosilactobacillus (L.) fermentum* CRL1446, isolated from goat cheese, improved the metabolic and oxidative status of mice with metabolic syndrome [12]. In ruminant production systems, there is evidence that FA can act as a promoter of animal growth [11,13]. Therefore, the increase in FA in silages, through inoculation with LAB positive for FAE activity (FAE+), can exert these beneficial effects on animals to which they are supplied. This has been recently evaluated in dairy goats [14]. In addition, this methodology will imply significantly lower costs when compared with the use of HA as chemical additives. 

To date, FAE+ LAB evaluated as silage inoculants are derived from vegetal sources [15,16,17]. There is evidence that animal-derived LAB can dominate the fermentation of forages and are more likely to improve animal production performance after consumption [18].

Based on these facts, we hypothesized that inoculating corn silage with potentially probiotic FAE+ LAB from goat feces can improve the conservation of the forage, its digestibility, and/or its antioxidant properties. The objective of this work was to isolate FAE+ LAB from goat feces, evaluate their potential probiotic properties in vitro, and study their aptitude as corn silage inoculants.

## 2. Materials and Methods

### 2.1. Isolation of FAE+ LAB from Goat Feces

Fifteen fecal swab samples were obtained from adult Creole goats in the northwest region of Argentina (Rincón de Quilmes, Tafí del Valle, Tucumán, Argentina). The three herds sampled (five samples each), each one on a different day during the fall season, belonged to small local farmers with an extensive production system, who did not apply any medical treatment for at least 6 months before the sampling date. Swabs transported in a refrigerated LATP medium (composition per L: peptone from bovine meat 15 g, tryptone 10 g, yeast extract 10 g, agar 7.5 g, Tween^®^ 80 1 mL) were then thoroughly mixed in phosphate buffer (pH 7.0), and appropriate dilutions were plated by surface-spreading on a Rogosa medium (Oxoid, Basingstoke, Hampshire, UK). After 48 h of incubation at 37 °C (5% CO_2_), colonies were picked and re-streaked for isolation. Isolates that were rod-shaped, positive to Gram stain, negative to catalase reaction, and nonmotile were tested to identify the presence of FAE activity according to Donaghy et al. [19]. Minor modifications of the technique included the use of cultures in the late stationary phase (16 h at 37°C, 5% CO_2_) and inoculation in 6 mm wells using 20 µL of bacterial cell suspensions (CSs). CSs were prepared after two cycles of centrifugation at 10,000× *g* and washing using 0.1 M phosphate buffer (pH 7.0). *L. fermentum* CRL1446 was used as the positive control in all assays [20]. Clear halos of FAE+ isolates were registered and measured after 48 h of incubation at 37 °C in aerobic conditions. These FAE+ strains were identified and deposited in the ETC collection (CERELA-CONICET, San Miguel de Tucumán, Tucumán, Argentina), and a code number was assigned. Storage was held at −80 °C on a LEL-glycerol medium (composition per L: dehydrated skim milk 100 g, yeast extract 50 g, glucose 10 g, glycerol 200 mL). Activation of strains before each assay was performed using 2% (*v*/*v*) inoculums on an MRS broth (Oxoid™, Basingstoke, Hampshire, UK) medium incubated at 37 °C for 16 h for three consecutive times.

### 2.2. Molecular Identification and Phylogenetic Analysis of Strains

Sequence analysis of 16S rRNA and four different housekeeping genes was employed to discriminate and identify the closely related strains from this study. Primer sequences are summarized in Table 1. PCR amplification conditions were as follows: 5 min heating at 94 °C, followed by 30 cycles of 30 sec denaturation at 94 °C, 30 sec annealing at 50 °C, and 1 min 30 sec extension at 72 °C, followed by a final extension step of 5 min at 72 °C. DNA amplicons were purified using a GenElute™ PCR Clean-Up Kit (Sigma-Aldrich, St Louis, MO, USA) for further sequencing. Chromosomal DNA was isolated according to Azcárate-Peril and Raya [21]. The 16S rRNA gene sequence was amplified by PCR using universal primers [22] and sequenced (ABI/Hitachi^®^ Genetic Analyzer 3130, Applied Biosystems, Foster City, CA, USA). Sequence alignments and similarity were performed using free open-source UGENE Software (Unipro, Novosibirsk, Russia) [23]. The 16s rRNA sequences obtained were initially compared with the nucleotide database of the National Centre of Biotechnology Information [24]. Additionally, the sequences of the housekeeping genes obtained in this study (access IDs are shown in Supplementary Table S1) were aligned with the type strains obtained from GenBank using CLUSTALX software, version 2.1 (Conway Institute, UCD, Dublin, Ireland) [25]. The phylogenetic trees were constructed using the PHYLIP neighbor-joining [26] and PhyML maximum-likelihood methods [27], and bootstrap analysis was performed with 100 replicates [28]. The sequence distance matrix for each gene was calculated using the Hamming dissimilarity algorithm with the F84 model [29] and drawn by UGENE software. 

### 2.3. General Probiotic Characterization

Isolated strains were evaluated according to the general characterization of lactobacilli (growth at 45 °C, 15 °C, pH 3.0, and 4% or 10% NaCl) in MRS broth for 24 h. The autoaggregation index and bacterial adhesion to n-hexadecane (the hydrophobicity index) were evaluated [31]. Simulated gastrointestinal tract (GIT) conditions [32], using a sequential incubation for 120 min in artificial gastric juice (pH 3.0) followed by 120 min in intestinal juice (0.3% bile salt concentration), were used to estimate the survival (%) of these strains and therefore their potential to exert probiotic benefits in the gut. Gelatinase [33], hemolytic [34], and lecithinase [35] activities were tested by agar diffusion methods in order to detect potentially virulent characteristics. These assays were performed at least in duplicate. 

### 2.4. FAE Activity Quantification at 37 °C

The method described by Yue et al. [36] was adapted to quantify the FAE activity present in the CS of the isolated strains. A 100 µM methyl ferulate (MF, Sigma-Aldrich, St Louis, MO, USA) solution in 0.1 M sodium phosphate buffer (pH 7.0) was used as the substrate. A mixture of the substrate and CS (2:1) was incubated in a water bath for 15 min at 37 °C. The reaction was stopped in an ice bath, and the tubes were centrifuged at 10,000× *g* for 1 min. The optical density of 300 µL of the supernatant was determined on a UV–visible microplate reader at 340 nm (TECAN Infinite M200 Pro^®^, Männedorf, Switzerland). The FAE activity was calculated using a standard curve of the substrate (0–83.3 µM). Blanks containing the substrate and sterile buffer were prepared and subjected to the same procedure. The dry weight (DW) of each CS was determined in a moisture analyzer (Ohaus^®^ MB35, Parsippany, NJ, USA) at 105 °C. One unit of specific FAE activity (U g^−1^) was defined as the amount of the CS that hydrolyzes 1 nm of MF per minute per g cells (in DW basis). 

To validate this method, the results were compared by means of a correlation analysis with the values obtained by HPLC quantification of ferulic acid (FA) released from the same substrate according to Mukdsi et al. [20]. Briefly, a 10 mM methanolic solution of the substrate was prepared and added to the CS in a 1:1 proportion. The reaction was incubated for 18 h at 37 °C, stopped by acidification using acetic acid, and, after proper preparation, analyzed by HPLC as stated in the reference technique using a reverse-phase C-18 column (ReproSil-Pur ODS, 3.5 µm, 250 × 4.6 mm; Dr Maisch GmbH, Ammerbuch, Germany).

### 2.5. Effect of pH and Temperature on FAE Activity

To assess the effect of the key variables on these enzymes, the incubation temperature was modified to 18 °C or the pH of the reaction buffer was lowered to 6.0 (the substrate in the 0.1 M phosphate buffer), 5.0, or 4.0 (the substrate in the 0.1 M acetate buffer). The results in these variable conditions were compared with those obtained at pH 7.0, 37 °C (standard conditions, StdC). The results for each variable condition were analyzed as U g^−1^ and also transformed to relative FAE activity (RU) considering StdC as 100% [RU= (U g^−1^ at variable condition × 100)/Mean U g^−1^ at StdC]. 

### 2.6. Growth in Maize Soluble Fraction Medium (MSFM)

Chopped (~1 cm) sweet corn stover (Zea mays var. SC 5148, Starke Ayres^®^, Johannesburg, SOU) was mixed at 20% (FM *w*/*v*) with distilled water for 15 min at 50 °C in constant agitation (Thermomix^®^, Vorwerk, Wuppertal, Germany) and then filtered through eight layers of medical gauze [37]. The obtained liquid medium (MSFM) was autoclaved at 115 °C for 20 min. To test the ability of the selected strains to grow in it, an inoculation rate was designed to achieve 10^4^ CFU mL^−1^ at the beginning of the incubation period, for which appropriate amounts of the CS of each strain were used. Tubes were statically incubated at 30 °C in aerobic conditions for 72 h. Growth was monitored by pH determination at 6, 12, 24, 48, and 72 h and viable LAB counts and reducing sugar content at 48 h. The pH was determined using a digital potentiometer (Altronix^®^ TPX I, Brooklyn, NY, USA), and the results were expressed as ∆pH (initial pH—*x* h pH). Viable LAB counts were determined by spreading of adequate dilutions on MRS agar plates and incubation at 37 °C for 48 h. Remaining reducing sugars (RSs) were determined by the Somogyi–Nelson method [38]. Independent experimental units for measuring the results at each time of incubation were used. 

### 2.7. Maize Mini Silo Experiment

#### 2.7.1. Inoculants, Forage, and Silage Preparation

The CSs of ETC150, ETC175, ETC187, and CRL1446 strains were used as silage inoculants. Whole-plant maize hybrid Pioneer R48 (418 g DM kg fresh matter (FM)^−1^, crude protein 51, NDF 613, and acid detergent fiber (ADF) 309 g kg DM^−1^) was manually harvested and chopped using a portable piece of equipment (JF MAXXIUM™, JF Máquinas Agrícolas Ltd., Itapira, SP, Brazil) to an average particle size of 4 cm. Forage was separated in three batches of 4 kg for each treatment and sprayed to reach an inoculation rate of 1 × 10^6^ CFU g FM^−1^ using 3 mL of each inoculant kg FM^−1^. A control group (UN, uninoculated) was prepared by inoculation of the same amount of the sterile buffer. Hand mix was performed for 3 min, after which the forage from each batch was ensiled in respective 3 ± 0.1 kg mini silos made of polyethylene plastic bags (Agrinplex^®^, Venados Manufactura Plástica, Martínez, BA, Argentina) in appropriate size. These had two sides heat-sealed and the upper and lower sides closed by torsion and a tied knot, resembling the techniques applied by small local farmers [39]. Mini silos were incubated at ambient temperature (18 ± 4 °C, mean ± SD) for 100 days, after which they were opened and analyzed as follows.

#### 2.7.2. Microbial Analysis

A 15 g sample of each silage was diluted in 135 mL of sterile phosphate buffer (pH 7.0) and processed in a laboratory blender (Stomacher™ 400, Seward, London, UK) during 60 seg at maximum speed. Appropriate dilutions in the same buffer were prepared and surface-plated on different media: plate count agar (PCA) for total mesophilic bacteria (TMB), MRS agar for LAB, and Sabouraud dextrose agar (SDA) for yeast and molds. PCA and MRS were supplemented with 100 mg L^−1^ cycloheximide. SDA was supplemented with 40 mg L^−1^ gentamicin and 100 mg L^−1^ ampicillin. Incubation time, oxygen, and temperature conditions were as follows: PCA 72 h, 30 °C, aerobic; MRS 48 h, 37 °C, 5% CO_2_; SDA 96 h, 20 °C, aerobic. Results are expressed as Log CFU g FM^−1^.

#### 2.7.3. pH and Chemical Analysis

A 30 g sample was diluted with distilled water (1:10), processed in a mixer for 60 sec, and then filtered through four layers of medical gauze to obtain an aqueous extract (AqE). The pH was immediately determined in this AqE using a digital pH meter, and a subsample of 1 mL was mixed with 0.25 mL of 60% (*v*/*v*) trichloroacetic acid solution, incubated for 15 min, and centrifuged in order to follow a deproteinization protocol. Samples where then filtered (0.22 µm, white GSWP, 25 mm; Millipore Corp., Burlington, MA, USA). From this fraction, HPLC analysis was performed to detect and quantify lactate, acetate, propionate, butyrate, and ethanol on a Knauer Wellchrom© system (Berlin, Germany) equipped with a RI detector K-2301 using an ion-exclusion column (Rezek ROA-Organic Acid H+^©^, 8 µm, 300 × 7.8 mm; Phenomenex, Torrance, CA, USA). A 20 µL sample was injected and an isocratic linear solvent gradient of 5 mM H_2_SO_4_ was run as an eluent at a flow rate of 0.6 mL min^−1^. The quantities of the detected compounds were determined from a regression curve of each standard. Total volatile acids are the sum of the detected quantities of lactate, acetate, propionate, and butyrate. Ferulic acid content was quantified as previously described (Section 2.4). AqE was also used for the quantification of total phenolic compounds (TPCs) by the Folin–Ciocalteu method as previously described [40], adapted to a 96-well microplate assay: 100 µL of nondiluted AqE was mixed with 10 µL of a Folin–Ciocalteu reagent (Sigma-Aldrich, St Louis, MO, USA) diluted 1:2 in distilled water, and then 40 µL of 15.9% Na_2_CO_3_ was added. The rest of the procedure was performed as in the cited method to express the results as µg of gallic acid equivalent (GAE) per mL of AqE. Free radical scavenging activity using 2,2-diphenyl-1-picrylhydrazyl (DPPH, Sigma-Aldrich, St Louis, MO, USA) was determined according to Tian et al. [41], including minor modifications: AqE was used nondiluted, a methanolic 0.2 mM solution of DPPH was used as a reagent, and mixtures were centrifuged for 10 min at 5000× *g* before transferring a 200 µL aliquot to a microplate. 

#### 2.7.4. Compositional and Digestibility Analysis

Samples were processed in the Laboratorio de Forrajes y Nutrición Animal (Instituto Nacional de Tecnología Agropecuaria EEA-Santiago del Estero) according to standardized procedures for the determination of compositional fractions, including DM (at 65 °C, not corrected for volatile compounds), ash, crude protein, acid detergent fiber (ADF), and neutral detergent fiber (aNDF, expressed inclusive of residual ash) [42]. Digestibility measures at 48 h of incubation were performed via Daisy II^®^ (ANKOM technology, Macedon, NY, USA) using rumen liquid from donors receiving a diet of alfalfa hay and corn.

### 2.8. Statistical Calculations

Studies were performed in duplicate in three independent assays, except for 2.7. The data obtained were analyzed using Infostat/L^®^ 2019 for Windows (Universidad Nacional de Córdoba, Córdoba, Argentina) [43]. Graphics were designed using GraphPad Prism^®^ version 8.0 for Windows (GraphPad Software, San Diego, CA, USA).

## 3. Results

### 3.1. Isolation of FAE+ LAB from Goat Feces

Twelve LAB-compatible isolates were obtained from goat feces; the rest (≈90%) were mainly coco-shaped bacteria and, in a minority of cases (≈2%), catalase-positive bacilli. Only three rod-shaped LAB, one from each of the herds sampled, showed FAE activity on an agar plate assay. They were medium- to long-sized rods that produce white, translucent colonies on MRS agar plates and were named ETC150, ETC175, and ETC187. Halos were clearly different between them: while control strain CRL1446 shows a sharp 11 mm Ø halo (mean value), ETC150 and ETC175 exhibit a diffuse 9 mm Ø halo, and ETC187 exhibits a diffuse 15 mm Ø halo noticeable even before 6 h of incubation. 

### 3.2. Molecular Identification of FAE+ Strains and Phylogenetic Analysis

**Figure 1:**
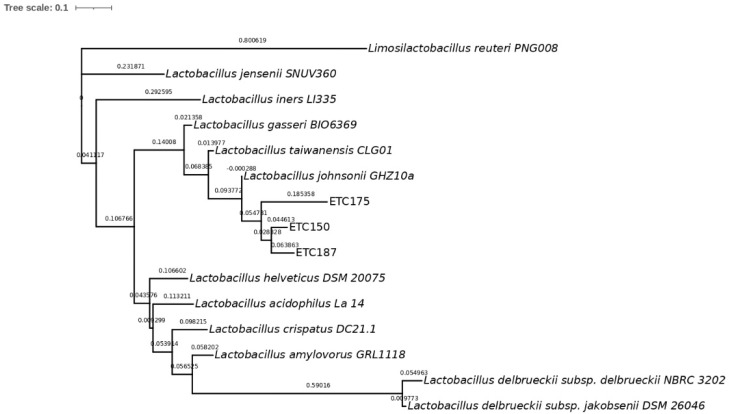
Maximum-likelihood tree with *pheS* gene sequences. Bar, 10% nucleotide substitutions.

**Figure 2:**
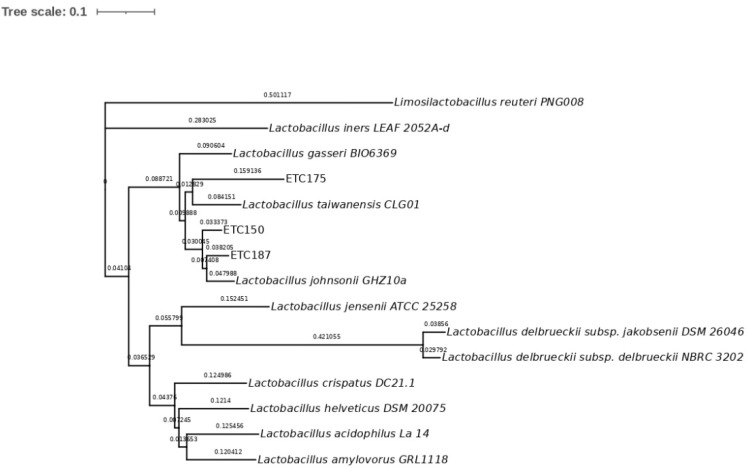
Maximum-likelihood tree with *gyrB* gene sequences. Bar, 10% nucleotide substitutions.

**Figure 3:**
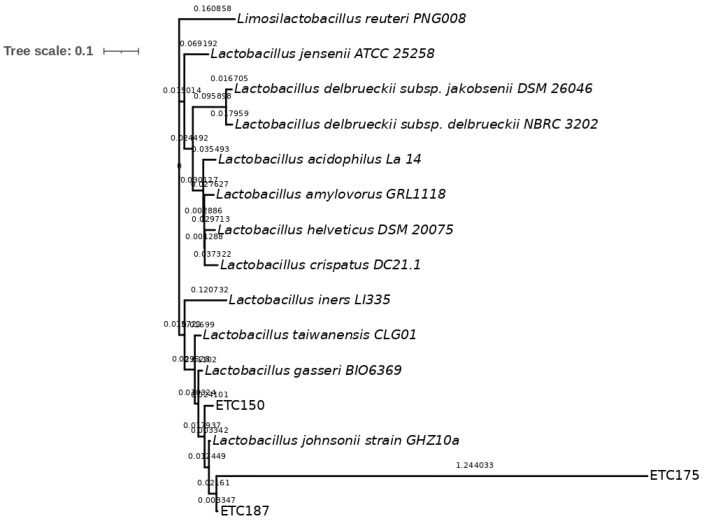
Maximum-likelihood tree with *tuf* gene sequences. Bar, 10% nucleotide substitutions.

**Figure 4:**
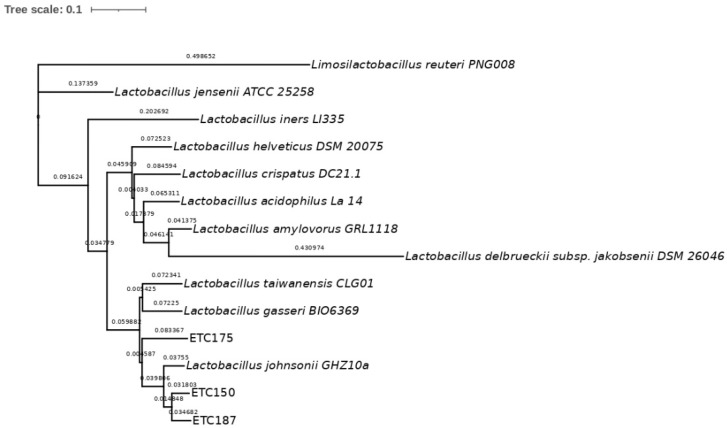
Maximum-likelihood tree with *pyrG* gene sequences. Bar, 10% nucleotide substitutions.

To identify the isolated strains ETC150, ETC175, and ETC187, a phylogenetic analysis was conducted. The 16S rRNA gene sequences of the isolated strains showed a high similarity with three different strains from this group, *L. johnsonii*, *L. taiwanensis*, and *L. gasseri*. The percentage of the similarity of ETC150 with the three reference strains was 99.56%. For the ETC175 strain, the similarity was 98.48% with *L. johnsonii*, whereas it was 98.26% with both *L. taiwanensis* and *L. gasseri*; and for the ETC187 strain, it was 99.35% with *L. johnsonii* and *L. taiwanensis*, and was 98.49% with *L. gasseri* (Supplementary Table S1). To better discriminate between these closely related strains, sequence alignment of housekeeping genes was performed. The selected genes were *pheS*, coding for the phenylalanine-tRNA ligase alpha subunit; *gyrB*, coding for the DNA topoisomerase (ATP-hydrolyzing) subunit B; *tuf*, coding for the elongation factor Tu; and *pyrG*, coding for the CTP synthase. The sequences of the selected housekeeping genes of 12 members of the *L. acidophilus* group collected from the NCBI database were included in the analysis. The multilocus sequence analysis (MLSA) approach performed to obtain the phylogenetic characterization of the isolated strains showed, as expected, a higher level of discrimination between them. Although these genes are highly conserved, they showed a higher degree of variability between closely related bacteria belonging to the *L. acidophilus* group than the 16S rRNA gene. The percentages of the identity of the isolated strains with the 12 members of the *L. acidophilus* group ranged as follows: from 50.3% to 96.99% for the *pheS* gene (Supplementary Table S2), from 68.27% to 98.05% for the *gyrB* gene (Supplementary Table S3), from 37.9% to 97.2% for the *tuf* gene (Supplementary Table S4), and from 64.24% to 96.39% for the *pyrG* gene (Supplementary Table S5). The gene that retrieved the highest percentages of identity was *gyrB*, with values above 98%. The phylogenetic analysis of the ETC150 strain allowed identifying it as *L. johnsonii*. The percentage of identity obtained with the *pheS* gene was 96.99% (Supplementary Table S1). The phylogenetic tree shows the strain clustering with *L. johnsonii* (Figure 1). The analysis and tree construction conducted with *gyrB* showed a percentage of identity of 98.05% (Figure 2). For the *tuf* gene, the percentage of identity was 96.84% (Figure 3) and was 95.81% when analyzing *pyrG* (Figure 4). The same identification was obtained for the ETC187 strain, obtaining percentages of identity of 95.03% with *pheS* (Figure 1), 98.05% with *gyrB* (Figure 2), 97.20% with *tuf* (Figure 3), and 96.39% with *pyrG* (Figure 4). For the ETC175 strain, the phylogenetic analysis with the *pheS*, *gyrB*, *tuf*, and *pyrG* genes did not allow a complete identification of it since the percentages of identity were below 90% for most of the analyzed genes, except for *pyrG*, which showed a slightly higher percentage of similarity of 90.2%. Additionally, the *tuf* gene sequence obtained for the ETC175 strain was not long enough to allow a proper identification. Although further characterization is needed for this strain to fully identify it, interestingly, the highest levels of identity were shown with the ETC150 and ETC187 strains from this study, identified as *L. johnsonii*, when analyzing both *pheS* and *pyrG* (Supplementary Tables S2 and S4, Figure 2 and Figure 4). However, when analyzing the *gyrB* gene, it is more closely related to *L. taiwanensis* (Supplementary Table S2, Figure 2). 

### 3.3. General Probiotic Characterization

Isolated strains were able to grow in a 4% NaCl-MRS medium and unable to grow at 15 °C or in a 10% NaCl-MRS medium, and a weak increase in turbidity was detected when they were incubated at 45 °C or in a pH 3.0 MRS (Supplementary Table S6). Medium (30–60%) values of autoaggregation were detected for all strains, as well as hydrophobicity. Simulated GIT conditions allowed a 90 ± 1.2% survival for ETC187 and 77 ± 1.3 and 75 ± 2% for ETC150 and ETC175, respectively. Alpha hemolysis was detected for all strains, which is caused by the acidification of the medium. Lecithinase and gelatinase tests were negative in all cases.

### 3.4. FAE Activity Quantification at 37 °C

The data obtained in three independent assays showed strong consistency for each strain (*p* < 0.0001, Table 2). The substrate hydrolysis rate was 78 ± 1% for the ETC187 strain and 43 ± 2%, 29 ± 3%, and 28 ± 3% for the CRL1446, ETC150, and ETC175 strains, respectively. An acceptable coefficient of determination (R^2^ = 0.976) was obtained between the results found using this method (considering µM of the substrate consumed by the CS after 15 min) and the HPLC quantification of free FA (µM) after 18 h of incubation (Supplementary Table S7). The correlation between the halo’s diameter in the diffusion agar method and FAE activity quantification using the spectrophotometric method described in this work (R^2^ = 0.976) or by HPLC quantification of FA (R^2^ = 0.990) was high. 

### 3.5. Effect of pH and Temperature on FAE Activity

**Figure 5:**
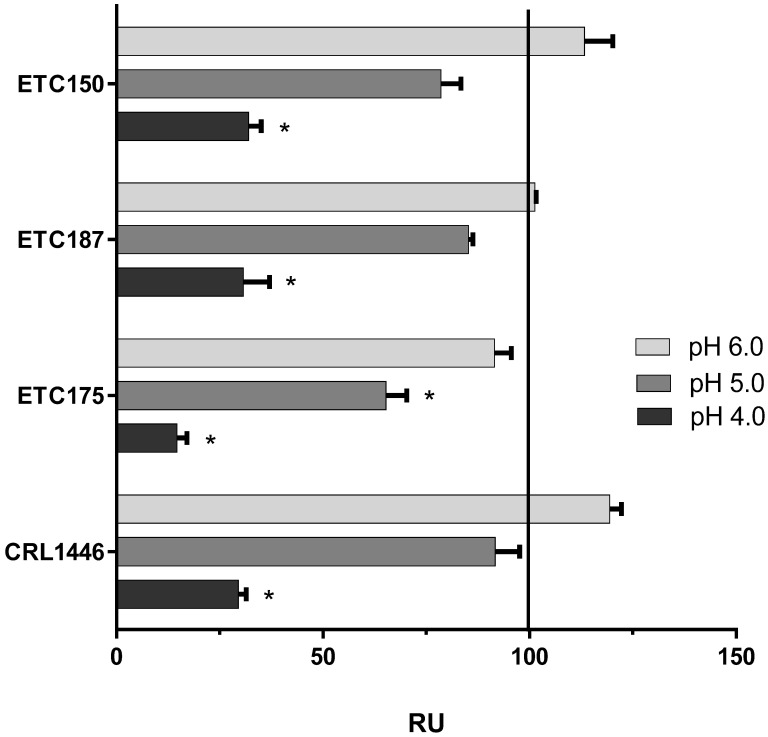
Effect of pH on FAE activity at 37 °C. RU= relative FAE activity (mean ± SE) at different pH conditions for each strain, considering mean U g^−1^ at pH 7.0 as 100% (black line, standard condition, StdC). An * indicates statistical difference (*p* < 0.05) in comparison with results at StdC for each strain.

Quantification of FAE activity at 18 °C is shown in Table 2. This temperature condition significantly decreased the FAE activity in all strains except ETC187; this is likely due to the high U g^−1^ of this strain. The effect of lower pH is shown in Figure 5. The lowest activity values were detected at pH 4.0 (RU = 26.7 ± 8.1) for all strains. Only ETC175 suffered a significant reduction of activity at pH 5.0. No statistical difference was detected between StdC and pH 6.0, although a tendency to higher U g^−1^ at this pH was seen for CRL1446 and ETC150. Regardless of pH and temperature, ETC187 showed the highest activity values, followed by CRL1446 (477.67 ± 15.38 and 274 ± 15.73, respectively).

### 3.6. Growth on MSFM

The obtained medium had an average pH of 5.6 ± 0.05 and an RS content of 30.2 ± 1.4 mmol glucose equivalent L^−1^, was fairly translucent, and had a small quantity of fine precipitate that could be easily dissolved by agitation. No significant growth was detected by pH measures at 6 h of incubation, for which this time point was excluded from analysis. After 72 h, no differences could be detected when compared with 48 h of incubation for which 48 h was set as the end point. The results are presented in Table 3. pH measures indicated a higher growth rate for ETC187 and CRL1466 at 12 and 24 h of incubation, while ETC187 presented the lowest pH at the end of the incubation when compared with the rest of the strains. The RS content at 48 h followed the pH determination pattern, but CFU counts only showed a lower growth for ETC175 when compared with the rest of the strains.

### 3.7. Maize Mini Silo Experiment

The density obtained in silages was 142 ± 12 kg DM m^3 −1^ at the beginning of incubation, and no significant variation was detected between experimental groups. Determinations made in silages after 100 days of incubation are presented in Table 4. Weight loss by gas production was significantly higher in UN silages than in the rest of the experimental groups. pH was significantly lowered by inoculation with ETC175, and lactate concentrations were higher in these silages when compared with those in CRL1446 silages. Butyrate and propionate were below the lower limit of detection in all samples analyzed. The yeast population tended to be lower in ETC187 silages than in UN silages (*p* = 0.062). Molds were not detected in any of the samples (<100 CFU g FM^−1^). DM loss was significantly reduced by ETC150 and CRL1446 inoculation. The ADF content was significantly diminished by ETC150, ETC187, and CRL1446 inoculation when compared with UN silages. DMD was significantly (*p* < 0.05) increased by CRL1446 by an average of 9.6%. The ferulic acid content was higher in AqE from ETC175-inoculated silages when compared with that from UN silages. The effect on the TPC content was also strain-dependent: ETC187-inoculated silages had lower content when compared with the silages inoculated with ETC150. DPPH scavenging activity was not altered by strain inoculation.

## 4. Discussion

### 4.1. Isolation of FAE+ LAB from Goat Feces

The isolation of lactobacilli from goat gut was intended to contribute with high FAE+ activity strains that are safe to add to a ruminant diet, as previous reports showed relatively low FAE+ activity for LAB isolated from silage and other vegetal sources [44]. Moreover, it pretended to evaluate the potential of nonsilage-originated LAB to act as inoculants [18,37]. There are a few studies published about the fecal microbial composition of goat gut [42,45] and, to the best of our knowledge, a single previous report on the isolation of LAB from this niche [46]. 

### 4.2. Molecular Identification of FAE+ Strains and Phylogenetic Analysis

*L. johnsonii*, *L. gasseri*, and *L. taiwanensis* are a compact cluster in the *L. acidophilus* complex, presenting difficulties for the distinct allocation of new isolates within species [30,47]. Species identification of isolated strains in this work, given on the basis of 16S rRNA sequence analysis, was insufficient, as identical and ≥97% identity scores were detected with different species, in contrast to what Kullen et al. [22] proposed using these primers, but in accordance with other reports, where the sequence similarity of a range of housekeeping genes was employed to identify the closely related strains [30]. *L. johnsonii* is a widely known probiotic species mostly isolated from mammalian gut [48], which, to the best of our knowledge, has not been tested as a silage inoculant. The growth characteristics of the isolated strains are partially in accordance with previous reports for this species [30,47], as inconsistent results have been registered by these authors. *L. taiwanensis* is a recently described distinct species from a silage isolate [47], after which it was also identified in a rat’s intestinal content [30]. Although these species are not usually described in silages, closely related species, such as *L. acidophilus* and *L. amylovorus*, were recently reported [17]. The FAE+ LAB employed as silage inoculants in experimental conditions belong to the species *Lentilactobacillus buchneri* [7,49,50], *Levilactobacillus brevis* [6,51], and *Limosilactobacillus fermentum* [52], among others, which are all heterolactic. This type of fermentative pattern is beneficial for some crops, but in many cases, combination or single homolactic inoculants are needed. It is especially important to consider that the latter type of inoculants is mainly predominant during the first stage of the fermentation process, while pH is less acidic and esterases can more efficiently exert their activity. To the best of our knowledge, this is the first report about the use of homolactic *L. johnsonii* strains as silage inoculants, whether considering FAE+-producing strains or not. 

### 4.3. General Probiotic Characterization

Probiotics are defined as “live microorganisms that, when administered in adequate amounts, confer a health benefit on the host” [53]. In livestock production systems, probiotics improve not only health but also production parameters such as milk yield, growth rate, and feed conversion efficiency, among others [54]. The proposed mechanisms of action include enhanced digestion and absorption of nutrients by increasing a range of enzymatic activities in the GIT. Several types of microorganisms have been evaluated as ruminant probiotics, including LAB, exerting inconstant results [55]. It is generally accepted that the natural microbiota of the intended ecosystem should be used as a source of probiotics. Our work provides one of the few reports about potential autochthonous probiotics for goats. Draksler et al. [56] isolated and characterized LAB from goat feces, which were then included in a mixed probiotic preparation that improved several health markers in goats [46]. These researchers inoculated selected LAB in sugarcane silages to be administered to young goats, which, in some cases, showed a modified gut microbiota and increased feed efficiency as a consequence of the treatment [57]. When selecting beneficial microbes to include as feed additives, it is vital to assure that they are nonpathogenic. *L. johnsonii* is a generally regarded as safe (GRAS) species [58], and no expression of virulence factors was detected on plate assays, although further investigation should be performed to complete their safety assessment. 

The delivery of probiotics through silage inoculation, or the probiotic effect of silage inoculants, was proposed and briefly studied [59,60]. To achieve this, the microorganisms should be able to survive the fermentation process in the silage to exert their effect in the rumen environment or the gut after consumption. Han et al. [60] demonstrated that certain silage LAB species are also present in cow feces, and that the majority of LAB are eliminated in the rumen and not in the postruminal segments [60]. The rumen is a complex ecosystem to reproduce in experimental conditions and highly variable according to the current diet. Weinberg et al. [61] proposed a method and identified silage inoculants that can survive rumen conditions. This is a rational assessment for the proposed ruminant probiotics, although there is no consensus regarding its suitability, and it is not usually performed. The selection of potential beneficial microbes usually focuses on survival to nonruminant GIT in vitro conditions [54]. Although there is not a generally accepted survival range for cattle, sheep, or goat probiotics, human-directed probiotic experts recommend a minimum survival to assure an optimal dose of 6–7 log CFU g^−1^ achieving the gut [62]. Strains isolated in this work were able to resist the simulated conditions of GIT, as expected considering their isolation source. The probiotic effect of a FAE+ *L. plantarum* strain when used as an alfalfa silage inoculant to feed goats was recently reported [14].

### 4.4. FAE Activity Quantification and Effect of pH and Temperature

Quantification and characterization of FAE LAB activity are usually an expensive and time-consuming objective. This is mainly because LAB cinnamoyl esterases previously described are usually intracellular or cell-surface associated [63,64], in contrast to the soluble enzymes produced by microorganisms such as molds [65]. Therefore, to obtain these enzymes, preparation of cell-free extracts [19] or cloning and hetero-expression [17,66] are usually needed. HPLC quantification of released HA is, to the best of our knowledge, the only method previously described that allows the use of bacterial cell suspensions. Spectrophotometric methods are, in contrast, simple, inexpensive, and more suitable for a large number of samples and kinetic measurements [65], but they were only described for enzymatic solutions. The execution of this spectrophotometric method proposed in this work was fast and convenient. This adapted method implies significantly less amount of the synthetic substrate and provides faster results when compared with the HPLC quantification of free FA. An acceptable correlation was detected between both techniques. In light of the previous [17,19] and present results, it is possible that a good approximation to relative FAE can be made by halo measures in the agar plate assay. In order to compare between different techniques and reports, FAE activity is sometimes measured in terms of the percentage of the consumed substrate after incubation or the substrate-to-product conversion rate: this resulted in high FAE activity for the ETC187 strain and intermediate activity for the rest [64,66,67]. 

The effect of temperature on FAE activity, with lower values at temperatures below 30 °C, is consistent with previous reports [16], although Fritsch et al. [66] did not observe reduced activity around 20 °C in most of the studied lactobacilli species. The optimal pH for different lactobacilli strains was frequently reported as 6.4 or 6.5 [16,19], while our results indicated no significant differences between pH 6.0 and 7.0. Reduction in the enzymatic activity by acidification was also described by Ding et al. [16], with a RU of around 20% at pH 4.0. Fritsch et al. [66] reported even higher acidic susceptibility of FAE enzymes from several lactobacilli strains. This evidence shows the importance of fast-growing strains that dominate the first stage of the fermentation process in silages, although some hydrolysis can still be effective in the stable phase of this process.

### 4.5. Growth on MSFM

Saarisalo et al. [37] proposed the preparation of forage extracts to evaluate potential silage inoculants, which included a long extraction process (120 min), filtering through cellulose filters and the addition of glucose. This method or similar ones were applied by several researchers [68,69,70]. The modifications introduced in this work provide a simplification of the technique, do not imply prolonged incubation at high temperature (which can alter certain substrates or enzymes), and avoid the retention of nutrients in the cellulose filter. A stable medium that was able to support the growth of most of the studied strains up to nearly 9 Log CFU mL^−1^ was obtained. This is similar to values observed after 16 h of incubation in an MRS medium (2% *v/v* inoculum) except for ETC175 (in which CFU counts on MRS are significantly higher; data not shown).

### 4.6. Maize Mini Silo Experiment

Silages were prepared at laboratory scale according to procedures used by goat farmers in the local area [39]. Silages compacted by manual methods or rudimentary-assisted, as small farmers usually do, reach suboptimal densities. Therefore, the preservation of forage is diminished [71], but improvements of the technique are hard to access due to many factors, including low financing access. DM recovery in similar corn mini silos [72], inoculated or not with several additives, was analogous to that observed in the UN group from our study. In our trial, inoculation with ETC150 and CRL1446 improved this important parameter (by 7.35% and 6.7%, respectively), and all treatments reduced gas losses. The latter parameter might indicate a dominance or beneficial alteration of inoculated LAB during the first stage of ensilage. Although ETC175 induced fermentative changes, such as lower final pH, and higher lactic acid and FA concentrations, no statistically significant effect was detected in the compositional or degradability analysis when compared with the UN group. Improved nutritional composition was observed in silages inoculated with the other two *L. johnsonii* strains isolated. Reduction in the ADF content was previously reported in maize silages by combined *L. buchneri* inoculation with fibrolytic enzymes [49], while it was detected for some FAE+ LAB inoculation in alfalfa silages [16,52]. NDFD was increased by *L. buchneri* FAE+ when applied to corn [7,73] or barley silage [3]. When measured in situ, NDFD was increased by the inoculation of different FAE+ LAB in perennial ryegrass [6]. In the present work, no effect on this parameter nor in the aNDF content was observed, as it was previously reported for similar treatments [6,14,16]. On the other hand, DMD was significantly increased (10%) by inoculation with CRL1446. Lynch et al. [49] reported a 2–4% DMD (measured at 24 h) increase in corn silages inoculated with combined fibrolytic enzymes–homolactic LAB–*L. buchneri* PTA-6138 but observed no differences in DMD at 48 h. In this case, the DMD of the UN group was similar to that observed in this work. Kang et al. [7] detected a 4% increase in this parameter (measured in situ) by the inoculation of the same strain, with higher values in the UN group when compared with our results. Similarly, FAE+ *L. fermentum* 17SD-2 reduced ADF and aNDF contents in alfalfa silages, but digestibility measures were not provided [52]. It is likely that the effects of FAE+ LAB on cell wall digestibility are strain-dependent, but forage differences, regarding not only species and varieties but also the maturity stage, are to be considered [74]. 

Silages are usually a poor source of antioxidants when compared with the rest of the usual ingredients in ruminant diets for which some interventions are tested in order to alleviate the oxidative stress in the animals and therefore enhance their health and production efficiency [41]. In this work, antioxidant determinations were performed in an AqE from silages. FA concentrations were around 10-fold lower than those reported in *Pennisetum sinese* silage extracts [75] but around 30-fold higher than those provided in co-ensiled corn stalk and potato pulp [76] or sorghum silages [77]. The higher FA concentration in ETC175-inoculated silages can be beneficial, as a previous report indicated an improvement in meat quality by the addition of 30 mg. kg^−1^ of FA to the diet of steers [13]. Interestingly, no correlation could be detected between FA concentration, TPC, and DPPH results in our experimental silages in contrast to previous reports on corn silages treated with fibrolytic enzymes [78] or alfalfa silages treated with FAE+ LAB [14]; however, in many cases, different types of determinations and different extraction methods were performed. It is proposed that FA reacts with increasing scavenging activity, but this could not be detected in the present experiment [14]. The TPC analyzed in this work was generally lower than that previously reported for corn silages [78], which, in that case, correlated with the amount of fibrolytic enzymes applied. The TPC content was negatively correlated with the digestibility measures by Taboada et al. [79], as observed in the CRL1446-inoculated group, but not in the rest of the experimental groups of this work.

Overall, it can be stated that FAE activity quantification and growth on MSFM were not able to predict the relative outcome of the evaluated strains when applied in a whole-plant corn mini silo experiment. Besides showing better growth performance in MSFM and the highest FAE activity, the ETC187 strain did not evidently improve silage fermentation in the present study when compared with the ETC150 and CRL1446 strains. As it is known, few publications studying the selection of silage inoculants are available [80,81], and a systematic, evidence-based process has not been consensually adopted. The radical difference in crop composition (due to species but also ambient conditions), complex microbial matrix of the ensiled crops, and diversity of ensiling techniques, among other multiple factors, contribute to the difficulties in anticipating the outcome of a certain silage inoculation and, therefore, the ability to predict it through in vitro liquid systems. Nevertheless, these screening methods are a rational approach to optimize the selection process. Our results are consistent with those of previous authors [69,81], who evidenced that silage inoculant aptitude is strain, not species, specific. 

## 5. Conclusions

Three *L. johnsonii* FAE+ strains (ETC150, ETC175, and ETC187) were isolated from goat feces and briefly studied regarding their potential probiotic aptitude, FAE activity, and growth ability in a maize-derived medium. These LAB showed intermediate to high FAE activity and high resistance to simulated GIT conditions. The inoculation of these strains in maize mini silages, including an experimental group inoculated with the human probiotic *L. fermentum* CRL1446, resulted in improved forage conservation for all treatments, but the nutritional profile was especially enhanced by the ETC150 and CRL1446 strains. These modifications in low-packing-density silages might derive in a significant increase in production efficiency, especially for small farmers who depend on low-quality forages and/or lack ensiling machinery. Furthermore, it is possible that the application of these strains as silage inoculants constitutes a probiotic-delivery strategy, enhancing production parameters not only as a consequence of forage preservation but also through health-improving mechanisms. This is the first report on the use of FAE+ *L. johnsonii* strains as corn silage inoculants in experimental conditions, and the results obtained encourage further studies. 

## Figures and Tables

**Table 1:** Primers used in phylogenetic analysis of isolated strains.

Primer	Sequence	Fragment Size (bp)	Reference
pheS F	5′-AAACTAGGGTGGTACCGCGA-3′	398	This study
pheS R	5′-GCTTTGAACCTAAATGTCCTTCAC-3′		This study
gyrB F	5′-TCACATTTCTGCTGGAACGAT-3′	458	This study
gyrB R	5′-CTACAGAAGCACCAACACCGT-3′		This study
pyrG F	5′-TTATGTTACYGAYGATGGTAC-3′	908	Sarmiento Rubiano et al. [30]
pyrG R	5′-ACCACGWGTACCAAAACCAC-3′		[30]
tuf F	5′-ATGGCAGAAAAAGAACATTACG-3′	1176	[30]
tuf R	5′-AGTAACYTGACCRGCACCAAC-3′		[30]

**Table 2:** Effect of temperature on Ferulic acid esterase (FAE) activity at pH 7.0.

Temperature (°C)	Strain				SEM	*p*-Value
	ETC150	ETC187	ETC175	CRL1446		
**37**	218 ^Ba^	591 ^c^	211 ^Ba^	330 ^Bb^	9.5	<0.0001
**18**	168 ^Aa,b^	513 ^c^	106 ^Aa^	216 ^Ab^		
SEM	13.5					
*p*-value	<0.0001					

FAE activity expressed as mean U g^−1^. Data were analyzed by means of two-way ANOVA procedure. Interaction between variables was not statistically significant (*p* > 0.05). Different superscript indicates statistically different results considering strain variable (lowercase letter) or temperature (capital letter). SEM: pooled standard error of means (*n* = 3).

**Table 3:** Growth of FAE+ strains in MSFM at 30 °C.

Item ^1^	Time (h)	ETC150	ETC175	ETC187	CRL1446	SEM	*p*-Value
∆pH	12	0.2 ^a^	0.2 ^a^	0.6 ^b^	0.5 ^b^	0.02	<0.001
	24	0.8 ^a^	0.6 ^a^	1.6 ^b^	1.4 ^b^	0.06	<0.001
	48	2.12 ^a^	2.29 ^b^	2.71 ^d^	2.46 ^c^	0.03	<0.0001
RS	48	21 ^b^	23.9 ^b^	7.3 ^a^	19.6 ^b^	1.12	<0.01
∆Log CFU mL^−1^	48	4.72 ^b^	3.62 ^a^	4.63 ^b^	4.79 ^b^	0.05	<0.01

^1^ Means of ∆pH (initial pH—pH at x h), reducing sugars (RS, mmol glucose equivalent L^−1^), and CFU counts (48 h Log CFU mL^−1^—initial Log CFU mL^−1^) are shown. Independent units were used for each incubation period studied. Data were analyzed by means of one-way ANOVA procedure followed by Tukey’s test for each time of incubation and each measure of growth. Different superscript letter indicates statistically different results (*n* = 3).

**Table 4:** pH, microbial, chemical, and nutritional measures of corn silages uninoculated (UN) or inoculated with FAE+ strains, after 100 days of incubation.

Item	UN	ETC150	ETC175	ETC187	CRL1446	SEM	*p*-Value	
pH	3.83 ^a,b^	3.86 ^b^	3.77 ^a^	3.85 ^b^	3.82 ^a,b^	0.01	0.02	*
Gas loss (%)	9.8 ^b^	4 ^a^	3.5 ^a^	3 ^a^	2.3 ^a^	0.06	<0.01	**
*Microbial populations, Log UFC g FM^*−1*^*								
Lactic acid bacteria	5.7	6.2	5.9	5.3	5.8	0.35	0.598	ns
Total mesophilic bacteria	5.0	5.3	5.4	5.4	6.6	0.2	0.053	t
Yeasts	4.8	3.3	3.3	2.7	3.6	0.35	0.062	t
*Fermentation products, g kg DM^*−1*^*								
Lactate	39.1	36.1	40.9	38.1	30.3	1.38	0.07	t
Acetate	19.8	14.4	15.6	17.3	15.8	1.29	0.83	ns
Total volatile acids	59	51	57	55	46	4.8	0.45	ns
Ethanol	1.0 ^a,b^	0.2 ^a^	0.2 ^a^	0.1 ^a^	3.75 ^b^	0.5	<0.05	ns
*Compositional and nutritional analysis, g kg DM* ^*−1*^								
DM loss	94 ^b^	20.5 ^a^	85.5 ^b^	71 ^b^	26.5 ^a^	7.32	<0.01	**
Crude protein	45	47	47	46	52	0.97	0.22	ns
aNDF	568	528	548	552	530	12	0.43	ns
NDFD	327	294	317	302	289	10	0.17	ns
ADF	269 ^c^	226 ^a^	255 ^b,c^	235 ^a,b^	231 ^a^	5.54	<0.01	**
DMD	607 ^a^	689 ^a,b^	631 ^a,b^	672 ^a,b^	703 ^b^	18	<0.05	*
*Antioxidant determinations*								
Ferulic acid (g kg DM^−1^)	135 ^a^	144 ^a,b^	211 ^b^	139 ^a,b^	95 ^a^	15	<0.01	**
DPPH scavenging activity (%)	85	86	88	88	84	1.6	0.82	ns
TPC (mg Eq AG 100 g^−1^)	51 ^b,c^	52 ^c^	48.6 ^a,b^	46.4 ^a^	47.8 ^a^	0.5	<0.01	**

DM: dry matter. FM: fresh matter. aNDF: neutral detergent fiber, expressed inclusive of residual ash. NDFD: in vitro degradability of NDF. ADF: acid detergent fiber. DMD: in vitro degradability of DM. TPC: total phenolic compounds. All values shown are means (*n* = 3). Different superscript letter indicates statistically different results (*p* < 0.05). ns (not significant): *p* ≥ 0.1; t (tendency): *p* < 0.1; *: *p* < 0.05. **; *p* < 0.01.

## Supplementary Materials

The following supporting information can be downloaded at: https://www.mdpi.com/article/10.3390/microorganisms10091732/s1; Table S1: % identity matrix of the partial 16S RNAr sequences between isolated and reference strains. Table S2: % identity matrix of the *pheS* gene sequences between isolated and reference strains. Table S3: % identity matrix of the *gyrB* gene sequences between isolated and reference strains. Table S4: % identity matrix of the *tuf* gene sequences between isolated and reference strains. Table S5: % identity matrix of the *pyrG* gene sequences between isolated and reference strains. Table S6: General probiotic characterization of isolated strains. Table S7: Correlation of quantification methods for FAE activity using bacterial cell suspensions.
