# Supplementary material for: Ferulic Acid Esterase Producing Lactobacillus johnsonii from Goat Feces as Corn Silage Inoculants
Source: Microorganisms. 2022 Aug 27;10(9):1732. doi: 10.3390/microorganisms10091732 (PMC9500823; doi:10.3390/microorganisms10091732)
Supplement: Supplementary file 1 [file microorganisms-10-01732-s001.zip › Table S1.pdf]

Supplementary Table S1. % Identity matrix of the partial 16S RNAr sequences between isolated and reference strains.

Accession ID	Strain	*L. johnsonii* **GHZ10a**	*L. taiwanensis* **CLG01**	*L. gasseri* **BIO6369**
		CP062068.1	CP059276.1	WBOA01000001.1
MT579619	**ETC150**	99.56	99.56	99.56
MT579620	**ETC175**	98.48	98.26	98.26
MT579621	**ETC187**	99.35	99.35	98.49
