# Supplementary material for: Ferulic Acid Esterase Producing Lactobacillus johnsonii from Goat Feces as Corn Silage Inoculants
Source: Microorganisms. 2022 Aug 27;10(9):1732. doi: 10.3390/microorganisms10091732 (PMC9500823; doi:10.3390/microorganisms10091732)
Supplement: Supplementary file 1 [file microorganisms-10-01732-s001.zip › Table S2.pdf]

Supplementary Table S2. % Identity matrix of the *pheS* gene sequences between isolated and reference strains.

Divergence	ETC150	ETC175	ETC187	*Limosilactobacillus reuteri* PNG008	*Lactobacillus delbrueckii* subsp. *jakobsenii*	*Lactobacillus delbrueckii* subsp. *delbrueckii*	*Lactobacillus jensenii* SNUV360	*Lactobacillus iners* LI335	*Lactobacillus johnsonii* GHZ10a	*Lactobacillus taiwanensis* CLG01	*Lactobacillus gasseri* BIO6369	*Lactobacillus crispatus* DC21.1	*Lactobacillus helveticus* DSM 20075	*Lactobacillus acidophilus* La-14	*Lactobacillus amylovorus* GRL1118
ETC150	100.00	83.84	93.28	50.30	56.63	56.02	64.46	60.24	96.99	86.75	88.25	68.67	68.07	71.08	67.77
ETC175	83.84	100.00	84.08	52.27	58.31	57.70	61.33	60.12	83.38	85.80	83.69	69.18	70.09	71.90	69.18
ETC187	93.28	84.08	100.00	51.86	59.63	59.01	65.84	60.87	95.03	89.13	89.13	68.01	68.01	71.74	69.25
Limosilactobacillus reuteri PNG008	50.30	e	51.86	100.00	59.03	58.83	62.56	61.13	63.13	62.56	61.51	61.13	62.66	61.70	61.51
Lactobacillus delbrueckii subsp. *jakobsenii*	56.63	58.31	59.63	59.03	100.00	99.24	68.76	62.19	66.67	66.86	66.76	70.67	69.33	68.76	71.24
Lactobacillus delbrueckii subsp. delbrueckii	56.02	57.70	59.01	58.83	99.24	100.00	68.67	62.10	66.19	66.76	66.29	70.29	68.86	68.29	70.86
Lactobacillus jensenii SNUV360	64.46	61.33	65.84	62.56	68.76	68.67	100.00	72.19	74.19	73.81	73.43	73.24	72.95	73.43	72.19
Lactobacillus iners LI335	60.24	60.12	60.87	61.13	62.19	62.10	72.19	100.00	74.38	74.67	74.10	70.57	72.10	72.86	70.95
	96.99	83.38	95.03	63.13	66.67	66.19	74.19	74.38	100.00	89.81	89.71	78.76	80.76	80.38	78.67

Lactobacillus johnsonii GHZ10a															
Lactobacillus taiwanensis CLG01	86.75	85.80	89.13	62.56	66.86	66.76	73.81	74.67	89.81	100.00	90.57	79.33	80.48	79.43	79.24
Lactobacillus gasseri BIO6369	88.25	83.69	89.13	61.51	66.76	66.29	73.43	74.10	89.71	90.57	100.00	79.90	81.62	79.90	79.52
Lactobacillus crispatus DC21.1	68.67	69.18	68.01	61.13	70.67	70.29	73.24	70.57	78.76	79.33	79.90	100.00	85.33	85.52	85.62
Lactobacillus helveticus DSM 20075	68.07	70.09	68.01	62.66	69.33	68.86	72.95	72.10	80.76	80.48	81.62	85.33	100.00	86.57	87.05
Lactobacillus acidophilus La-14	71.08	71.90	71.74	61.70	68.76	68.29	73.43	72.86	80.38	79.43	79.90	85.52	86.57	100.00	88.19
Lactobacillus amylovorus GRL1118	67.77	69.18	69.25	61.51	71.24	70.86	72.19	70.95	78.67	79.24	79.52	85.62	87.05	88.19	100.00
