# Supplementary material for: Ferulic Acid Esterase Producing Lactobacillus johnsonii from Goat Feces as Corn Silage Inoculants
Source: Microorganisms. 2022 Aug 27;10(9):1732. doi: 10.3390/microorganisms10091732 (PMC9500823; doi:10.3390/microorganisms10091732)
Supplement: Supplementary file 1 [file microorganisms-10-01732-s001.zip › Table S3.pdf]

Supplementary Table S3. % Identity matrix of the *gyrB* gene sequences between isolated and reference strains.

Divengence	*Limosilactobacillus reuteri* PNG008	*Lactobacillus delbrueckii* subsp. *jakobsenii*	*Lactobacillus delbrueckii* subsp. *delbrueckii*	*Lactobacillus iners* LEAF 2052A-d	ETC175	*Lactobacillus taiwanensis* CLG01	*Lactobacillus gasseri* BIO6369	*Lactobacillus johnsonii* GHZ10a	ETC150	ETC187	*Lactobacillus jensenii* ATCC 25258	*Lactobacillus acidophilus* La-14	*Lactobacillus amylovorus* GRL1118	*Lactobacillus helveticus* DSM 20075	*Lactobacillus crispatus* DC21.1
Limosilactobacillus reuteri PNG008	100.00	65.73	65.83	68.02	68.27	69.12	68.54	68.75	69.86	69.70	69.80	67.97	68.18	67.66	67.81
Lactobacillus delbrueckii subsp. *jakobsenii*	65.73	100.00	99.75	67.69	67.39	72.43	72.27	72.27	71.25	70.86	74.44	72.32	74.77	73.70	74.11
Lactobacillus delbrueckii subsp. *delbrueckii*	65.83	99.75	100.00	67.74	67.63	72.43	72.38	72.38	71.50	71.11	74.59	72.32	74.82	73.75	74.16
Lactobacillus iners LEAF 2052A-d	68.02	67.69	67.74	100.00	74.10	75.59	75.03	75.23	75.43	75.06	75.66	75.38	75.23	75.64	75.08
ETC175	68.27	67.39	67.63	74.10	100.00	87.23	86.05	85.82	88.05	87.56	73.72	73.10	73.57	75.48	74.29
Lactobacillus taiwanensis CLG01	69.12	72.43	72.43	75.59	87.23	100.00	88.67	90.19	91.95	91.97	77.35	81.58	81.12	81.27	79.90
Lactobacillus gasseri BIO6369	68.54	72.27	72.38	75.03	86.05	88.67	100.00	90.50	92.68	92.21	77.91	81.48	80.41	81.98	80.20
Lactobacillus johnsonii GHZ10a	68.75	72.27	72.38	75.23	85.82	90.19	90.50	100.00	98.05	98.05	77.10	81.98	81.53	82.09	81.07

ETC150	69.86	71.25	71.50	75.43	88.05	91.95	92.68	98.05	100.00	98.53	76.31	77.07	75.85	78.54	77.32
ETC187	69.70	70.86	71.11	75.06	87.56	91.97	92.21	98.05	98.53	100.00	76.69	76.96	75.74	77.94	76.72
Lactobacillus jensenii ATCC 25258	69.80	74.44	74.59	75.66	73.72	77.35	77.91	77.10	76.31	76.69	100.00	77.71	78.43	77.66	79.40
Lactobacillus acidophilus La-14	67.97	72.32	72.32	75.38	73.10	81.58	81.48	81.98	77.07	76.96	77.71	100.00	86.87	86.51	84.73
Lactobacillus amylovorus GRL1118	68.18	74.77	74.82	75.23	73.57	81.12	80.41	81.53	75.85	75.74	78.43	86.87	100.00	86.06	86.46
Lactobacillus helveticus DSM 20075	67.66	73.70	73.75	75.64	75.48	81.27	81.98	82.09	78.54	77.94	77.66	86.51	86.06	100.00	86.51
Lactobacillus crispatus DC21.1	67.81	74.11	74.16	75.08	74.29	79.90	80.20	81.07	77.32	76.72	79.40	84.73	86.46	86.51	100.00
