# Supplementary material for: Ferulic Acid Esterase Producing Lactobacillus johnsonii from Goat Feces as Corn Silage Inoculants
Source: Microorganisms. 2022 Aug 27;10(9):1732. doi: 10.3390/microorganisms10091732 (PMC9500823; doi:10.3390/microorganisms10091732)
Supplement: Supplementary file 1 [file microorganisms-10-01732-s001.zip › Table S4.pdf]

Supplementary Table S4. % Identity matrix of the *tuf* gene sequences between isolated and reference strains.

Divergence	ETC175	*Limosilactobacillus reuteri* PNG008	*Lactobacillus delbrueckii* subsp. *jakobsenii*	*Lactobacillus delbrueckii* subsp. *delbrueckii*	*Lactobacillus iners* LI335	*Lactobacillus crispatus* DC21.1	*Lactobacillus acidophilus* La-14	*Lactobacillus helveticus* DSM 20075	*Lactobacillus amylovorus* GRL1118	*Lactobacillus jensenii* ATCC 25258	ETC150	ETC187	*Lactobacillus taiwanensis* CLG01	*Lactobacillus gasseri* BIO6369	*Lactobacillus johnsonii* GHZ10a
ETC175	100.00	42.65	41.91	41.91	42.65	40.44	40.44	41.18	41.18	40.44	37.90	41.88	41.91	41.91	41.18
Limosilactobacillus reuteri PNG008	42.65	100.00	80.44	80.18	81.02	83.29	82.45	82.70	82.96	83.96	82.78	83.30	85.22	85.31	84.97
Lactobacillus delbrueckii subsp. *jakobsenii*	41.91	80.44	100.00	99.66	82.79	88.16	88.16	88.50	88.66	86.06	83.14	83.66	85.39	85.81	86.06
Lactobacillus delbrueckii subsp. *delbrueckii*	41.91	80.18	99.66	100.00	82.79	87.99	87.99	88.33	88.50	86.23	83.05	83.57	85.31	85.73	85.98
Lactobacillus iners LI335	42.65	81.02	82.79	82.79	100.00	84.63	84.13	84.21	84.05	85.47	85.84	86.37	88.92	89.00	88.66
Lactobacillus crispatus DC21.1	40.44	83.29	88.16	87.99	84.63	100.00	95.30	96.64	96.73	88.83	86.74	87.36	89.50	90.09	89.59
Lactobacillus acidophilus La-14	40.44	82.45	88.16	87.99	84.13	95.30	100.00	96.56	96.89	89.84	85.66	86.10	88.41	89.08	88.50
Lactobacillus helveticus DSM 20075	41.18	82.70	88.50	88.33	84.21	96.64	96.56	100.00	97.40	88.92	86.29	86.91	89.08	89.34	89.17
Lactobacillus amylovorus GRL1118	41.18	82.96	88.66	88.50	84.05	96.73	96.89	97.40	100.00	89.34	86.02	86.64	88.66	89.34	88.92

Lactobacillus jensenii ATCC 25258	40.44	83.96	86.06	86.23	85.47	88.83	89.84	88.92	89.34	100.00	87.83	88.36	90.18	90.34	90.60
ETC150	37.90	82.78	83.14	83.05	85.84	86.74	85.66	86.29	86.02	87.83	100.00	98.00	95.13	95.13	96.84
ETC187	41.88	83.30	83.66	83.57	86.37	87.36	86.10	86.91	86.64	88.36	98.00	100.00	95.49	95.49	97.20
Lactobacillus taiwanensis CLG01	41.91	85.22	85.39	85.31	88.92	89.50	88.41	89.08	88.66	90.18	95.13	95.49	100.00	98.66	98.15
Lactobacillus gasseri BIO6369	41.91	85.31	85.81	85.73	89.00	90.09	89.08	89.34	89.34	90.34	95.13	95.49	98.66	100.00	98.15
Lactobacillus johnsonii GHZ10a	41.18	84.97	86.06	85.98	88.66	89.59	88.50	89.17	88.92	90.60	96.84	97.20	98.15	98.15	100.00
