# Supplementary material for: Ferulic Acid Esterase Producing Lactobacillus johnsonii from Goat Feces as Corn Silage Inoculants
Source: Microorganisms. 2022 Aug 27;10(9):1732. doi: 10.3390/microorganisms10091732 (PMC9500823; doi:10.3390/microorganisms10091732)
Supplement: Supplementary file 1 [file microorganisms-10-01732-s001.zip › Table S5.pdf]

Supplementary Table S5. % Identity matrix of the *pyrG* gene sequences between isolated and reference strains.

Divergence	*Limosilactobacillus reuteri* PNG008	*Lactobacillus delbrueckii* subsp. *jakobsenii*	*Lactobacillus jensenii* ATCC 25258	*Lactobacillus iners* LI335	ETC175	*Lactobacillus johnsonii* GHZ10a	ETC150	ETC187	*Lactobacillus taiwanensis* CLG01	*Lactobacillus gasseri* BIO6369	*Lactobacillus crispatus* DC21.1	*Lactobacillus helveticus* DSM 20075	*Lactobacillus acidophilus* La-14	*Lactobacillus amylovorus* GRL1118
Limosilactobacillus reuteri PNG008	100.00	62.48	68.98	65.54	64.32	65.79	64.24	64.32	67.02	66.52	67.29	66.60	66.85	65.61
Lactobacillus delbrueckii subsp. *jakobsenii*	62.48	100.00	68.89	68.09	69.70	71.05	68.45	69.35	71.53	72.27	73.70	73.40	73.89	75.86
Lactobacillus jensenii ATCC 25258	68.98	68.89	100.00	76.05	75.52	77.65	76.25	76.11	77.83	78.13	77.78	78.70	78.33	77.41
Lactobacillus iners LI335	65.54	68.09	76.05	100.00	78.55	79.48	78.00	78.21	78.42	77.68	79.44	78.58	78.64	76.85
ETC175	64.32	69.70	75.52	78.55	100.00	89.98	90.09	89.82	89.50	90.20	82.87	85.08	83.68	83.80
Lactobacillus johnsonii GHZ10a	65.79	71.05	77.65	79.48	89.98	100.00	95.81	96.39	91.31	90.14	83.40	83.77	82.96	82.47
ETC150	64.24	68.45	76.25	78.00	90.09	95.81	100.00	97.78	90.33	89.63	82.54	84.17	83.12	82.07
ETC187	64.32	69.35	76.11	78.21	89.82	96.39	97.78	100.00	90.09	89.51	82.40	83.80	82.98	82.05
Lactobacillus taiwanensis CLG01	67.02	71.53	77.83	78.42	89.50	91.31	90.33	90.09	100.00	91.56	83.51	83.88	83.45	83.08
Lactobacillus gasseri BIO6369	66.52	72.27	78.13	77.68	90.20	90.14	89.63	89.51	91.56	100.00	83.94	84.31	83.88	83.69
Lactobacillus crispatus DC21.1	67.29	73.70	77.78	79.44	82.87	83.40	82.54	82.40	83.51	83.94	100.00	90.06	89.51	89.88
Lactobacillus helveticus DSM 20075	66.60	73.40	78.70	78.58	85.08	83.77	84.17	83.80	83.88	84.31	90.06	100.00	90.49	89.81
Lactobacillus acidophilus La-14	66.85	73.89	78.33	78.64	83.68	82.96	83.12	82.98	83.45	83.88	89.51	90.49	100.00	91.23

Lactobacillus amylovorus GRL1118	65.61	75.86	77.41	76.85	83.80	82.47	82.07	82.05	83.08	83.69	89.88	89.81	91.23	100.00
