# Supplementary material for: Ferulic Acid Esterase Producing Lactobacillus johnsonii from Goat Feces as Corn Silage Inoculants
Source: Microorganisms. 2022 Aug 27;10(9):1732. doi: 10.3390/microorganisms10091732 (PMC9500823; doi:10.3390/microorganisms10091732)
Supplement: Supplementary file 1 [file microorganisms-10-01732-s001.zip › Table S6.pdf]

Supplementary Table S6. General probiotic characterization of isolated strains.

	ETC150	ETC175	ETC187
**Growth in MRS broth at/in**			
**15ºC**	-	-	-
**45ºC**	w	w	w
**4% NaCl**	+	+	+
**pH 3.0**	w	w	w
**Auto-aggregation (%)[1]**	37 ± 5*	30 ± 3	35 ± 5
**Hydrophobicity index (%)[1]**	35 ± 8	30 ± 5	31± 6
**Survival to simulated GIT (%)[1]**	77 ± 1.3	75 ± 2	90 ± 1.2

(-): Negative growth in tested conditions, ΔDO [DO_{24h}-$DO_{initial}$] ≤0.2; (+): Possitive growth. ΔDO≥0.5; w: week growth, ΔDO=0.21-0.49. [1]Calculated according to the reference techniques equations. (*): mean ± SD.
