# Supplementary material for: Ferulic Acid Esterase Producing Lactobacillus johnsonii from Goat Feces as Corn Silage Inoculants
Source: Microorganisms. 2022 Aug 27;10(9):1732. doi: 10.3390/microorganisms10091732 (PMC9500823; doi:10.3390/microorganisms10091732)
Supplement: Supplementary file 1 [file microorganisms-10-01732-s001.zip › Table S7.pdf]

Supplementary Table S7. Correlation of quantification methods for FAE activity using bacterial cell suspensions.

BL	**Spectrophotometric assay** MF reduction (μM)	**HPLC assay** FA (μM)	**Agar diffusion** Ø halo (mm)
ETC150	27	481	9.2
ETC175	25	527	9.0
ETC187	53	1090	15.5
CRL1446	35	646	11.6

**Spectrophotometric assay**	**HPLC assay**	**Agar diffusion**	**R^2**
MF reduction (μM)	FA (μM)		0.976
MF reduction (μM)		Ø halo (mm)	0.990
	FA (μM)	Ø halo (mm)	0.960

Average values (*n*=3) are shown.
